# Characteristics Analysis Reveals the Progress of *Volvariella volvacea* Mycelium Subculture Degeneration

**DOI:** 10.3389/fmicb.2019.02045

**Published:** 2019-09-03

**Authors:** Xiao Chen, Zheng Zhang, Xiaoxia Liu, Bo Cui, Wentao Miao, Weiwei Cheng, Fengyun Zhao

**Affiliations:** ^1^College of Food Science and Engineering, Gansu Agricultural University, Lanzhou, China; ^2^School of Food Science and Technology, Qilu University of Technology, Jinan, China; ^3^Yucheng People’s Hospital, Dezhou, China; ^4^College of Food and Bioengineering, Henan University of Science and Technology, Luoyang, China

**Keywords:** *Volvariella volvacea*, mycelial subculture, degeneration, biological traits, nutrients, early identification

## Abstract

*Volvariella volvacea* is a typical edible Basidiomycete with a high-temperature tolerance. It has a strong fibrinolysis capability and consumes abundant agricultural wastes. In agricultural cultivation, mycelial subculturing has been adopted, leading to serious strain degeneration. In this study, continuous mycelial subculturing of the common *V. volvacea* strain V971 (original strain recorded as M0) was performed in potato dextrose agar (PDA) medium. One generation of the strain was preserved every 3 months (90 days); thus, six generations of degenerated strains (M1–M6) were obtained after 18 months of mycelial subculturing. The original and degenerated strains were preserved in sterile paraffin liquid at room temperature (18–25°C). The biological traits and nutrients of M0 and M1–M6 were studied. The mycelial growth rate and biomass initially increased and then decreased as the degeneration progressed, reaching minimum levels of 0.041 ± 0.001 cm/h and 1.82 ± 0.25 g, respectively, at M6. Additionally, the polysaccharide, protein, polyphenol, flavone, total amino acid, and total mineral element contents of the strains decreased continuously, reaching minimum levels of 30.12 ± 3.12 g/100 g, 26.42 ± 2.1 g/100 g, 1.08 ± 0.05 g/100 g, 4.23 ± 0.21 g/100 g, 12.51 mg/g, and 398.05 mg/kg, respectively, at M6. The decolorization capability of *V. volvacea* in liquid medium supplemented with bromothymol blue and lactose reflected the degree of strain degeneration, with the capability weakening as the degeneration intensified. These results are highly significant for *V. volvacea* production. The mycelial characteristics during subculture-associated degeneration were described and provide an early identification method for *V. volvacea*’s degeneration.

## Introduction

Many edible mushroom species have been consumed as food and used as food flavoring for thousands of years. They are also an important drug source (Rai et al., 2005). However, strain degeneration restricts the development of the edible mushroom industry and decreases their biotechnological values (Magae et al., 2005). The preservation of edible mushroom strains is difficult because they are often characterized by the inability to form resistant propagules in pure cultures. Edible mushroom strains can only be preserved by serial transfer on agar with or without the addition of mineral oil (Voyron et al., 2009) or covered with sterile distilled water (Borman et al., 2006), or by cryopreservation and freeze-drying processes (Kitamoto et al., 2002; Palacio et al., 2017). Mycelial subculturing is a common technique used by growers. Generally, strains are subcultured every 3–4 months to maintain activity. However, the excessive subculturing of edible mushrooms may cause degeneration (Chen et al., 2017; Yin et al., 2017). Degenerated edible mushrooms are characterized by slim, fragile, and slow-growing mycelia (Magae et al., 2005; Kim et al., 2014).

*Volvariella volvacea* (Bull. ex. Fr.) Singer, also known as Chinese mushroom or straw mushroom, is an edible Basidiomycete that was introduced into China in the 18th century, and it was so valuable that it was often presented as a tribute to Chinese royalty (Chang, 1969, 1977). In 2010, the annual output of *V. volvacea* on the Chinese mainland was 330,000 tons, which accounted for more than 80% of the global output (Bao et al., 2013). *V. volvacea* is a typical edible straw mushroom with a high-temperature tolerance that preferentially grows at 30°C. Its fruiting bodies are popular with consumers owing to their taste and high nutrient contents. Moreover, *V. volvacea* contains many bioactive substances with medicinal values, such as anticancer-associated polysaccharides, immunosuppressive proteins, and immunoregulation-associated agglutinins (Mathew et al., 2008; Wu et al., 2011; Sun et al., 2014). Like other edible mushrooms, degeneration limits *V. volvacea* production and development (Sermkiattipong and Charoen, 2014). In agricultural cultivation, because of *V. volvacea*’s poor low-temperature resistance, it cannot be stored at low temperatures like other edible mushrooms (Guo et al., 2016). Growers prefer subculture storage for *V. volvacea*. The cultivation techniques (Ahlawat et al., 2011; Bao et al., 2013), breeding techniques (Zhao et al., 2010; Liu et al., 2011), and physiology (Diamantopoulou et al., 2016; Li et al., 2017) of *V. volvacea* have been investigated, but the serious consequences of strain degeneration have been overlooked. The degeneration of *V. volvacea* has been studied in China (Hu et al., 2015; Li and Wang, 2015); however, studies on the effects of mycelial subculturing on the characterizatics of edible mushrooms, specifically *V. volvacea*, have been limited.

The mycelia of edible mushrooms are rich in nutrients (Huang et al., 2006; Chiu et al., 2010) and are widely used in fermentation and medicinal fields (Mathew et al., 2008; Diamantopoulou et al., 2012; Zhang et al., 2017). Their agricultural and commercial values decrease along with the decreasing vitality of degenerated strains. However, many growers overlook the degeneration that occurs in the mycelial stage. Serious degenerative problems occur at the fruiting stage, which can result in huge economic losses (Magae et al., 2005). Thus, methods to identify degeneration early in *V. volvacea* strains should be investigated. In this study, mycelial subculturing was used to preserve *V. volvacea* strains. Moreover, the biological characteristics of, and nutrients in, degenerated mycelia were determined, and a simple method to detect *V. volvacea* strain degeneration was proposed. This study provides a theoretical basis for the early identification of degeneration in *V. volvacea* and other edible mushrooms.

## Materials and Methods

### Instruments, Chemicals, and Reagents

An LGJ-12 vacuum freeze-dryer was purchased from Songyuan Huaxing Technology Develop Co., Ltd. (Beijing, China). A TU-1901 spectrophotometer was purchased from Purkinje General Instrument Co., Ltd. (Beijing, China). An 835-50 automatic amino acid analyzer was purchased from Hitachi, Ltd (Tokyo, Japan), and a NoVAA400P flame atomic absorption spectrometer (F-AAS) was purchased from Analytik Jena AG Company (Jena, Germany). A FE20K electronic handheld pH meter was purchased from Mettler Toledo Company (Zurich, Switzerland). In the experiments, the ultrapure water used was produced from a Milli-Q integral water purification system (Millipore Corp., Billerica, MA, United States).

Bromothymol blue (BTB, cas#: 34722-90-2), lactose (cas#: 63-42-3), glucose (cas#: 50-99-7), rutin (cas#: 153-18-4), and gallic acid (cas#: 149-91-7) were purchased from Aladdin Bio-Chem Technology Co., Ltd. (Shanghai, China). The hollow cathode lamp and standards (purity ≥99%) of different mineral elements were purchased from Guobiao Testing and Certification Co., Ltd. (Beijing, China). All of the reagents used in the pretreatment of mycelial samples for automatic amino acid and F-AAS analyses were of guaranteed purity.

### Microorganisms, Media, and Culture Conditions

Commercial *V. volvacea* species (V971) in agricultural cultivation were purchased from the Edible Mushroom Research Institute, Jiangsu, China. In the experiments, all of the strains were cultured at 30°C if necessary. The potato dextrose broth (PDB) medium contained 200 g of fresh potato, 20 g of glucose, 1.0 g of KH_2_PO_4_, 1.0 g of MgSO_4_ ⋅ 7H_2_O, and 1,000 mL of distilled water. The potato dextrose agar (PDA) medium contained 1,000 mL of PDB and 20 g of agar. Liquid medium supplemented with BTB and lactose (LBL) contained 200 g of potato, 20 g of lactose, 2 g of NH_4_NO_3_, 1.5 g of KH_2_PO_4_, 0.5 g of MgSO_4_, 0.06 g of BTB, and 1,000 mL of distilled water at a pH of 7.0 (Magae et al., 2005).

The tip mycelia of *V. volvacea* were subcultured on PDA medium every 4 days. A 1-cm^2^ tip mycelial agar piece was transferred into the center of a fresh PDA plate and subcultured using the same method for another 18 months. Degenerated strains were collected from the last subculturing after 3 months (90 days) and individually stored in PDA slants. The original strain was recorded as M0, and the first generation of the degenerated strain was recorded as M1. Six generations of degenerated strains were prepared (M1–M6). Sterile liquid paraffin was injected into the PDA slant tubes, and the edible mushrooms were stored at room temperature (18–25°C).

For the detection of degeneration in *V. volvacea* strains, five pieces of 1-cm^2^ tip mycelial agar were inoculated into 100 mL LBL medium. Cultures were shaken for 8 days in the dark (30°C, 110 rpm). An additional five pieces of 1-cm^2^ PDA media without *V. volvacea* were used as the control group (CK). After 8 days, the media were centrifuged at 8,000 × *g* for 1 min, and the OD values of the supernatants were determined at 615 nm (Magae et al., 2005). The pH values of the LBL media were measured using an electronic handheld pH meter. The formula for calculating the decolorizing rate (expressed as a percentage) of LBL media was as follows:

DecolorizingrateofLBLmedium(%)

$$=\frac{\text{ODck}-\text{ODsample}}{\text{ODck}}\times 100\%$$

### Mycelial Growth Rate Assay

The mycelial growth rate was determined using the method of Diamantopoulou et al. (2012) with slight modifications. Briefly, the *V. volvacea* colonies radii were measured at 48 h after each inoculation. The growth rate of each generation of strains indicated the ratio of total *V. volvacea* strain colony radius at 90 d to 23 (the *V. volvacea* colony radius was measured 23 times over the 90-days period). The formula for the growth rate of each generation was as follows:

Growth⁢rate⁢of⁢mycelia⁢(cm/h)⁢

=the⁢total⁢V.v⁢o⁢l⁢v⁢a⁢c⁢e⁢a⁢colony⁢radius⁢in⁢  90⁢days23⁢×⁢48⁢h

### Mycelial Biomass Assay

The mycelial biomass of each generation of *V. volvacea* was determined using the method reported by Diamantopoulou et al. (2012) with slight modifications. Briefly, *V. volvacea* strains were activated twice on PDA medium, and five pieces of 1-cm^2^ tip mycelium agar were cultured in 100 mL of PDB medium for 8 days at 30°C. After the culture solution and agar pieces were removed, the mycelia were washed with deionized double-distilled water (ddH_2_O) three times. The mycelial biomass’ concentration was determined from the dry weight. Fungal mycelia were harvested from the vacuum freeze-dryer. Afterward, the mycelia were weighed, and each sample was tested 10 times.

### Mycelial Nutrient Assay

Vacuum freeze-dried mycelia (0.5 g) were pulverized and passed through a 60-mesh sieve. They were then carefully collected and placed in 10 mL 60% methanol solution (Rajauria et al., 2010). The mixture was processed at 100°C for 15 min and centrifuged at 5,000 × *g* for 15 min at 4°C to obtain the supernatant. Residues were extracted twice using the same method, and the supernatants were processed three times and mixed, to obtain the crude nutrient extract. The total polysaccharide content was determined using the phenol–sulfuric acid method. The standard curve was prepared with glucose as the standard, and the absorbance values of samples were detected at 490 nm (Zhou et al., 2014). The total flavone content was determined using NaNO_2_-Al(NO_3_)_3_-NaOH colorimetry. The standard curve was prepared with rutin as the standard, and the absorbance values of samples were detected at 510 nm (Ozsoy et al., 2008). The total polyphenol content was determined using the Folin–Ciocalteu reagent method. The standard curve was prepared with gallic acid as the standard, and the absorbance values of samples were detected at 760 nm (Mihai et al., 2012). The total protein content was tested using the Kjeldahl method. As described in the method, nitric organic substances were converted into ammonium salts, and ammonia was distilled to the standard acid solution. The results are presented in mg per 1 g of vacuum-freeze dried mycelia (Barbano et al., 1991).

A certain measured amount of vacuum freeze-dried mycelia was pulverized, passed through a 60-mesh sieve and placed into a hydrolysis tube. Next, 6 N HCl (10 mL) was added, and the tube was vacuum-packed and sealed. The mycelia were hydrolyzed at 110°C for 24 h. Subsequently, they were evaporated to dryness and diluted to the appropriate concentration. They were used to determine the contents of various amino acids in mycelia using an automatic amino acid analyzer. Test conditions for the analyzer were set as follows: chromatographic column diameter, 2.6 × 150 mm; ion exchange resin, Hitachi-2619 type; flow rate of buffer solution, 0.225 mL/min; flow rate of ninhydrin, 0.3 mL/min; pumping pressure of buffer solution, 80–120 kg/cm^2^; pumping pressure of ninhydrin, 15–35 kg/cm^2^; column temperature, 53°C; sample feeding rate, 50 μL; standard sample feeding rate, 3 nmol/50 μL; and nitrogen pressure, 0.28 kg/cm^2^.

Trace elements (Ca, Cu, Fe, K, Mg, Mn, Na, and Zn) were determined in each vacuum freeze-dried mycelial sample using F-AAS. The pretreatment of samples was based on the method reported by Li et al. (2011). Laboratory glassware was kept overnight in a 10% *v*/*v* HNO_3_ solution and then rinsed with deionized ddH_2_O. In total, 1 g of powdered sample was weighed into a 100-mL glass beaker. Then, 10 mL of HNO_3_ and 2 mL of H_2_O_2_ were added to the sample. The mixture was digested on a hot plate at low temperature for 2 h, and afterward, 15 mL of deionized water was added. Solutions were precisely transferred to 100-mL volumetric flasks and filled to the full volume with deionized ddH_2_O. The optical parameters of F-AAS used for mineral element determination experiments are shown in Supplementary Table S1.

### Statistical Analyses

All of the samples were analyzed in triplicate, unless otherwise specified. The results were expressed as means ± standard deviations (SDs) and subjected to an analysis of variance. The means were separated using Duncan’s multiple range test with the aid of SPSS version 22.0 software (SPSS Inc., United States). A heatmap was created using R software (v. 3.1.0).^1^ Variables included the amino acid and mineral element data, which were subsequently tagged as irrational values after the log-normalization generated in the R software.

## Results

### Mycelial Growth Rates of Different *V. volvacea* Strains

**FIGURE 1:**
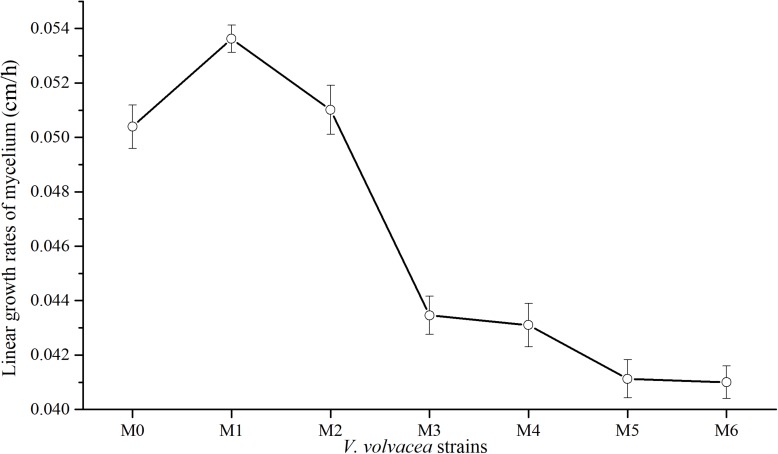
Mycelial growth rates of *V. volvacea* strains. Values represent the means ± SDs of triplicate samples.

Subcultures of *V. volvacea* mycelia were passaged for 18 months, and the kinetic behaviors of the tested *V. volvacea* strains were assessed on PDA medium. The obtained radial growth rates of M0–M6 were determined (Figure 1). The growth rates of *V. volvacea* mycelia significantly changed during the subculturing, reaching a maximum at M1 (0.0534 ± 0.001 cm/h) and sharply declining thereafter until M6 (0.041 ± 0.001 cm/h). The growth rates of the mycelia tended to be stable at M3 and reached their minimum values at M6, showing significant differences compared with M0 (0.0503 ± 0.001 cm/h; *P* < 0.05).

### Mycelial Biomasses of Different *V. volvacea* Strains

**FIGURE 2:**
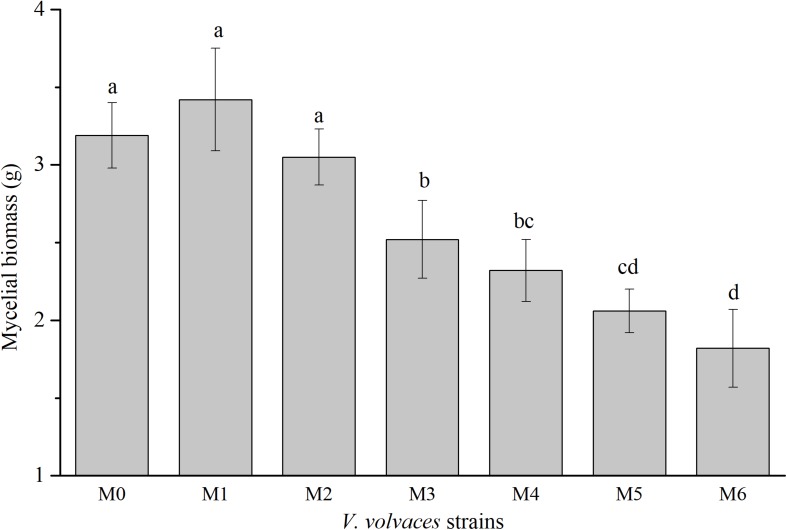
Dry mycelial biomasses of *V. volvacea* strains. Values represent the means ± SDs of triplicate samples. Different letters were significantly different (*P* < 0.05).

Subcultures of *V. volvacea* mycelia were passaged for 18 months on PDA medium, and mycelial biomasses of M0–M6 were determined (Figure 2). The mycelial biomasses of M0, M1, and M2 were 3.19 ± 0.21 g, 3.42 ± 0.33 g, and 2.62 ± 0.13 g, respectively, and there were no significant differences among the three groups (*P* > 0.05). However, the mycelial biomasses of M0, M1, and M2 were significantly different from those of other generations (M3–M6; *P* < 0.05). The maximum mycelial biomasses occurred at M1, while the minimum mycelial biomasses were achieved at M6 (1.82 ± 0.25 g).

### Polysaccharide, Protein, Flavone, and Polyphenol Contents in Different *V. volvacea* Strains

**FIGURE 3:**
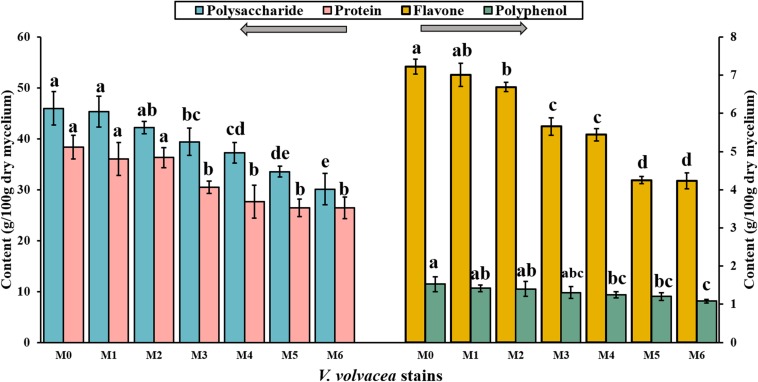
Polysaccharide, protein, flavone, and polyphenol contents in the dry mycelia of *V. volvacea* strains. Values represent the means ± SDs of triplicate samples. Different letters were significantly different (*P* < 0.05).

Subcultures of *V. volvacea* mycelia were passaged for 18 months on PDA medium, and the contents of polysaccharide, protein, flavone, and polyphenol compounds in M0–M6 were determined (Figure 3). M0 had the greatest nutrient values (polysaccharide, 46.01 ± 3.3 g/100 g; protein, 38.36 ± 2.32 g/100 g; flavone, 7.22 ± 0.2 g/100 g; and polyphenol, 1.53 ± 0.19 g/100 g). With the continuation of *V. volvacea* mycelial subculturing, the nutrient contents of the degenerated strains gradually decreased. The polysaccharide, protein, flavone, and polyphenol contents deceased to 30.12 ± 3.12 g/100 g, 26.42 ± 2.1 g/100 g, 4.23 ± 0.21 g/100 g, and 1.08 ± 0.05 g/100 g, respectively, at M6, which were significantly different compared with their values at M0 (*P* < 0.05).

### Changes in Amino Acid and Mineral Element Contents in Different *V. volvacea* Strains

**FIGURE 4:**
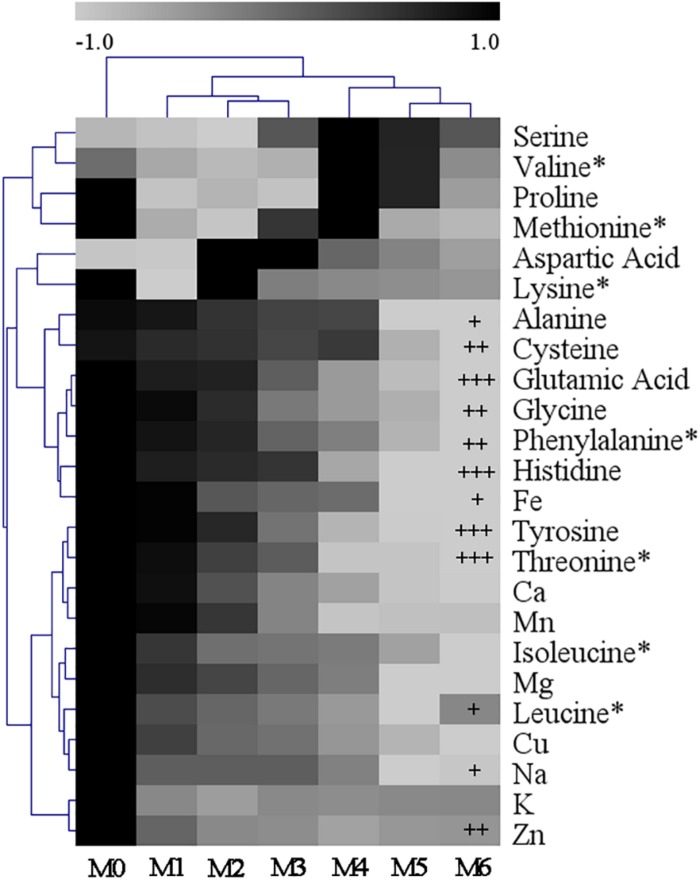
Heatmap showing the normalized content values of amino acids and mineral elements among the different *V. volvacea* strains. The normalized content values are depicted visually from black to gray; black represents the greatest value and gray the lowest value. The dendrogram distances represent the amino acids and mineral elements (left) based on their content values in the strains (top). *T*-tests were used to identify the differences between M0 and M6. +: *P* < 0.05; ++: *P* < 0.01; and +++: *P* < 0.001. ^∗^: essential amino acid.

Subcultures of *V. volvacea* mycelia were passaged for 18 months on PDA medium. The amino acid and mineral element contents in M0–M6 were determined and are shown in Supplementary Tables S2, S3, respectively. *V. volvacea* contained abundant and various amino acids and mineral elements. The total amino acid and mineral element contents in M0 were 12.51 mg/g and 398.05 mg/kg, respectively. However, they decreased continuously as the subculturing continued and reached 7.18 mg/g and 332.11 mg/kg, respectively, in M6. The amino acid and mineral element data were normalized and expressed as heatmaps (Figure 4). The glutamic acid, histidine, tyrosine, and threonine contents in M6 were significantly lower than those in M0 (*P* < 0.001). Additionally, the cysteine, glycine, phenylalanine, and Zn contents in M6 were significantly lower than those in M0 (*P* < 0.01). The greatest amino acid and mineral contents were found in M0. Although the contents decreased to some extent as the subculturing continued, not all of the contents had positive correlations with subculture time (e.g., aspartic acid, serine, lysine, and K). The arginine and tryptophan contents were not determined in this study (Supplementary Table S2).

### LBL Medium for Detecting the Degeneration of *V. volvacea* Strains

**FIGURE 5:**
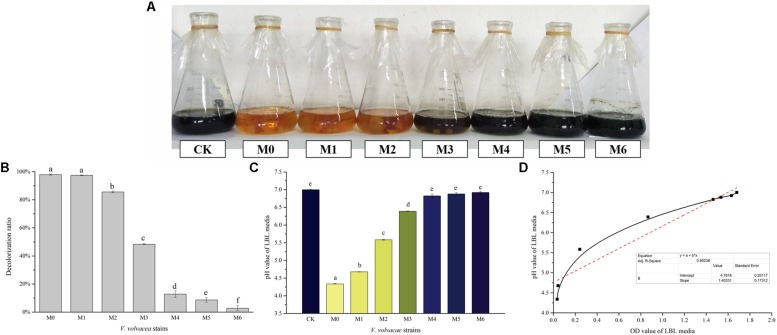
Characterization of LBL media inoculated with *V. volvacea* strains. **(A)** Visual colors of the LBL *V. volvacea* mycelial fermentation media. **(B)** Decolorization ratios of the LBL media. **(C)** pH values of the LBL media. **(D)** Correlation analyses between pH and OD values of the LBL *V. volvacea* mycelial fermentation media. Values represent the means ± SDs of triplicate samples. Different letters were significantly different (*P* < 0.05).

The decolorization capabilities of M0–M6 in LBL media were evaluated. Changes in visual color, decolorization ratios, and pH values were determined, and the results are shown in Figures 5A–C, respectively. As the degeneration of *V. volvacea* strains progressed, the decolorizing rate of the LBL media gradually weakened, and the color changed from orange (M0) to dark blue (M6, which was similar to CK) (Figure 5A). The decolorization ratios of M0 and M6 LBL media were 97.94% ± 0.42% and 2.84% ± 1.7%, respectively, showing a significant difference (*P* < 0.05) (Figure 5B). The pH values of M0, M6, and CK LBL media were 4.34 ± 0.01, 6.92 ± 0.03, and 7.00 ± 0.01, respectively. The pH values of M0 and M6 (or CK) LBL media were significantly different (*P* < 0.05), while the CK and M6 LBL media values showed no significant difference (*P* > 0.05; Figure 5C). In addition, positive correlations between pH and OD values were observed in LBL media inoculated with *V. volvacea* strains (coefficient of determination *R*^2^ = 0.902; Figure 5D), indicating the lower decolorization capabilities of the degenerated *V. volvacea* strains in LBL media.

## Discussion

Liquid paraffin can be used to store most fungi for a long time periods, even decades. After residual paraffin is removed from mycelia, normal strain growth can be recovered (Little and Gordon, 1967). Because the liquid paraffin-based storage techniques of edible mushroom strains require complicated operational environments and skillful technologies, growers often adopt simple subculturing processes, which cause continuous degeneration and great losses. Magae and Hayashi (1999) discovered two dsRNA elements and viral-like particles in the mycelia of *Flammulina velutipes* derived from a spontaneously brown-colored fruit body. They were not detected in the normal strains in fruiting-impaired degenerative isolates. Kim et al. (2014) reported that the symptoms of degenerated *F. velutipes* strains include slow vegetative growth, a compact mycelial mat, and few, or even no, fruiting bodies. Yin et al. (2017) artificially cultured the entomopathogenic mushroom *Cordyceps militaris* for six generations and observed changes during fruiting-body growth. The subcultured *C. militaris* strains began degenerating in the third generation, with incomplete fruiting-body growth beginning in the fourth generation. In the entomogenous fungi *Metarhizium anisopliae*, strains showed a reductions in, or the infertility of, spores after degeneration, accompanied with the development of villiform mycelia and changes in mitochondrial DNA (Wang et al., 2005). In this study, the symptoms of degenerated *V. volvacea* strains included slow mycelial growth rates and low mycelial biomasses, which are similar to the findings of a previous study (Sun et al., 2017). Additionally, the cultivation experiment revealed that, compared with M0, the biological efficiencies of M1–M3 significantly decreased, and the production cycles were significantly prolonged. The M4–M6 obtained by mycelial subculturing for 12–18 months lack the capabilities to produce fruiting bodies (unpublished data). These degradation-related characteristics are basically consistent with those of other edible mushrooms (Sun et al., 2017). The degeneration of edible mushrooms occurs frequently during agricultural cultivation. However, limited studies on degeneration resulting from the mycelial subculturing of edible mushrooms, especially *V. volvacea*, have been performed.

Basidiomycete’ hyphae grow continuously through the divisions of tip cells (Badalyan et al., 2004). The tip mycelia of Basidiomycetes have strong activity levels, and in the ectomycorrhizal basidiomycete *Amanita muscaria*, they are linked to the expression and transcriptional level of an α-tubulin gene. They also have a direct impact on the mycelial growth rate (Tarkka et al., 2006). In addition, Li et al. (2003) cultured tip mycelia of *Sclerotinia sclerotiorum* (vegetable fungi) on a PDA medium and found that the tip mycelia were highly stable. The tip mycelia reached a growth state and could generate a strong toxicity. Thus, the tip mycelia of *V. volvacea* were chosen for subculturing in this study. The mycelial growth rate can determine the growth-related vitality of *V. volvacea* mycelia. The greater the growth rate, the stronger the mycelial activity level (Mata and Savoie, 1998; Ahlawat et al., 2008). In this study, the growth rates of degenerated strains declined continuously (Figure 1), which might be the consequences of the reduced activity levels of *V. volvacea*. The mycelial biomasses of edible mushrooms can reflect their outputs, to some extent. Generally, the culture media used for *V. volvacea* were optimized based on mycelial biomass (Diamantopoulou et al., 2016). In this study, there was no significant difference in the mycelial biomass between M1 and M0 (*P* > 0.05), but there were significant differences in the mycelial biomasses between M2–M6 and M0 (*P* < 0.05) (Figure 2). Thus, the mycelial biomasses of degenerated strains decreased continuously as the subculturing continued, which might result in unsatisfying, and even a lack of, output from degenerated strains (Kim et al., 2014; Chen et al., 2017; Yin et al., 2017). Moreover, we discovered a similar trend between the mycelial growth rate and mycelial biomass as the subculturing continued. Therefore, the mycelial growth rate and mycelial biomass might be positively correlated.

*Volvariella volvacea* is rich in nutrients (Wang et al., 2009). Changes in nutrients are an important indication of *V. volvacea* degeneration. They not only represent the commercial and agricultural values of *V. volvacea* but also reflect the strength of internal biochemical reactions. In this experiment, the nutrients of M0 and M1–M6 were determined. The polysaccharide, protein, flavone, and polyphenol contents decreased continuously as the subculturing continued (Figure 3), and the effects of the subculturing were greatest on glutamic acid, histidine, tyrosine, and threonine levels (Figure 4). However, this trend was not observed for all nutrients, such as aspartic acid, serine, K, and Fe (Figure 4). Strain degeneration results in huge losses to the growers and presents a great challenge to breeders.

Edible mushrooms degrade cellulose, lignin, and other macromolecules in the medium into micromolecules for growth and development, which requires mutual conversions among sugars, proteins, amino acids, and other substances (Drewnowska and Falandysz, 2015). Valine and serine have strong inhibitory effects on the growth of mycelia (Kalac, 2009), and similar results were obtained here, with the valine and serine contents increasing from M1 to M4 (Figure 4). Alanine can effectively improve the absorption of nitrogen by mycelia and promote the formation of primordia and fruiting bodies (Meng et al., 2019). In this study, alanine showed a decreasing trend from M1 to M6, which may explain why *V. volvacea* could not produce fruiting bodies after 12 months of continuous mycelial subculturing. Mineral nutritional elements are the necessary activators or active agents in many enzyme reactions in *V. volvacea.* More than 50–70% of enzymes need to be activated by mineral elements to catalyze related biochemical reactions (Santos et al., 2015). Cu is located in the active site of laccase, which is helpful for xylogen degradation (Liu et al., 2015), and laccase in *V. volvacea* is correlated with fruiting body formation (Chen et al., 2003). Ca may contribute to the aggregative effect of enriched nutrient elements, and strains enriched with Ca may promote the carboxymethyl cellulase activity (Maijala et al., 1991). Zn is an essential element for the synthesis of tryptophan. When edible fungi lack Zn, the activity levels of superoxide dismutase in cells decreases (Bellomo et al., 2014). The fat content of a strain enriched with Fe significantly increase (Jedidi et al., 2017). In this study, the contents of eight mineral elements in the mycelia of *V. volvacea* gradually decreased from M1 to M6, which probably lead to the decreased activities of cellulase and other enzymes, contributing to the further degradation of *V. volvacea*.

In Japan, in 2005, Magae et al. (2005) identified the degeneration of *F. velutipes* based on color changes in the LBL medium. Degenerated strains have poor decolorizing capabilities in the LBL medium. Here, we reported that the decolorizing capability of the *V. volvacea* strains in LBL medium weakened as they degenerated (Figures 5A,B). Although this identification method is simple and easy-to-operate, the mechanism remains unknown. BTB in LBL media, a textile dye derivative often deployed as a pH indicator, which turns yellow in an acid environment, has been used as an indicator of free radical pollutants (Hoag et al., 2009). Hoag et al. (2009) demonstrated a decrease in the rate of BTB degradation as the amounts of free radicals increased. In this experiment, the changes in active substances (e.g., polyphenol, flavone, and mineral elements) in *V. volvacea* strains during degeneration were investigated (Figures 3, 4). The active substances could eliminate reactive oxygen species and inhibit lipid peroxidation (Ebrahimzadeh et al., 2010). As the degeneration progresses, the radical-scavenging capability decreases because the levels of these active components are reduced, which may lead to the lack of a significant color change in the media of degenerated strains. Moreover, during fermentation, organic acids, amino acids, and CO_2_ are produced by metabolic strains. These substances have high solubility levels in aqueous solutions and produce free hydrogen ions in water. In a medium with a low buffering capacity, the reaction results in a severe drop in pH (Figure 5C), which further affects the color of the LBL medium (Shen et al., 2004; Zhao et al., 2015). As the degeneration intensifies, the decolorizing rate of the LBL medium gradually decreased as the pH value of the fermentation liquid increased. In addition, the color changed from orange (M0) to brown (M3), and then to dark blue in M4–M6 (Figures 5A,D), which was consistent with cultivation experiments in which only M0–M3 could produce fruiting bodies (unpublished data). The experiment clearly showed that when the LBL medium turned dark blue, *V. volvace*a had degraded to the point where it was no longer suitable for production. This provides a method for the early identification of *V. volvacea* degeneration, which will help avoid economic losses caused by blind cultivation. Overall, the LBL medium assay, a simple colorimetric method, was highly efficient in identifying degenerated *V. volvacea* strains with poor nutrient levels and low decolorizing capabilities.

## Conclusion

As a typical edible straw mushroom with a high-temperature tolerance, *V. volvacea* cannot be stored under low temperature. Mycelial subculturing facilitates the degeneration of *V. volvacea* strains. In this study, the original generation (M0) and degenerated strains (M1–M6) of *V. volvacea* were acquired by subculturing mycelia for 18 months. The biological traits and nutrient contents of these strains were determined. As the subculturing continued, the growth rates and biomasses of *V. volvacea* mycelia initially increased and then decreased. The total polysaccharide, protein, polyphenol, flavone, amino acid, and mineral contents gradually deceased. The LBL medium was used to identify degenerated *V. volvacea* strains. The decolorizing capability of degenerated *V. volvacea* strains in LBL medium was weakened. This study discusses the degeneration of *V. volvacea* strains during subculturing and offers a feasible rapid identification method for *V. volvacea* strain degeneration.

## Author Contributions

ZZ and XC wrote the manuscript. ZZ and FZ designed the research. ZZ and XL performed the research. BC and WM provided experimental materials and equipment. XC and WC analyzed the data.

## Conflict of Interest Statement

The authors declare that the research was conducted in the absence of any commercial or financial relationships that could be construed as a potential conflict of interest.

## Footnotes

1 http://cran.cnr.berkeley.edu/
