# Supplementary material for: Characteristics Analysis Reveals the Progress of Volvariella volvacea Mycelium Subculture Degeneration
Source: Front Microbiol. 2019 Sep 3;10:2045. doi: 10.3389/fmicb.2019.02045 (PMC6733957; doi:10.3389/fmicb.2019.02045)
Supplement: Supplementary file 1 [file Table_1.DOCX]

**Supplementary material**

**Table S1** Optical parameters of F-AAS used in this study

| HCL type | wavelength (nm) | lamp currents (mA) | slit width (nm) | C_2_H_2_ flow (L/min) | air flow (L/min) |
| --- | --- | --- | --- | --- | --- |
| Ca | 422.7 | 6 | 0.5 | 2.0 | 8.0 |
| Cu | 324.8 | 3 | 0.5 | 1.8 | 8.0 |
| Fe | 248.3 | 8 | 0.2 | 2.0 | 8.0 |
| K | 766.5 | 5 | 0.5 | 1.9 | 8.0 |
| Mg | 285.2 | 4 | 0.5 | 1.6 | 8.0 |
| Mn | 279.5 | 5 | 0.4 | 1.9 | 8.0 |
| Na | 589.0 | 6 | 0.5 | 1.6 | 8.0 |
| Zn | 213.9 | 4 | 0.5 | 2.0 | 8.0 |

HCL: hollow cathode lamp.

**Table S2** Amino acid contents of dried mycelia of different *V. volvacea* strains

| amino acids (mg/g) | M0 | M1 | M2 | M3 | M4 | M5 | M6 |
| --- | --- | --- | --- | --- | --- | --- | --- |
| Aspartic Acid | 0.16±0.02 | 0.15±0.04 | 1.24±0.03 | 1.03±0.03 | 0.56±0.01 | 0.44±0.02 | 0.32±0.03 |
| Serine | 0.63±0.06 | 0.62±0.09 | 0.60±0.08 | 0.75±0.03 | 0.95±0.11 | 0.82±0.07 | 0.75±0.06 |
| Glutamic Acid | 0.38±0.04 | 0.32±0.00 | 0.32±0.01 | 0.27±0.03 | 0.21±0.02 | 0.18±0.04 | 0.12±0.02 |
| Glycine | 0.64±0.08 | 0.59±0.07 | 0.55±0.05 | 0.46±0.03 | 0.43±0.09 | 0.40±0.04 | 0.31±0.08 |
| Histidine | 0.50±0.05 | 0.42±0.05 | 0.41±0.07 | 0.39±0.07 | 0.23±0.02 | 0.15±0.06 | 0.13±0.04 |
| Alanine | 0.88±0.12 | 0.86±0.07 | 0.82±0.02 | 0.80±0.05 | 0.80±0.05 | 0.55±0.03 | 0.54±0.07 |
| Proline | 0.76±0.08 | 0.42±0.05 | 0.44±0.06 | 0.42±0.08 | 0.71±0.06 | 0.65±0.14 | 0.48±0.09 |
| Cysteine | 0.30±0.05 | 0.28±0.03 | 0.27±0.04 | 0.25±0.07 | 0.26±0.00 | 0.16±0.04 | 0.05±0.01 |
| Tyrosine | 1.88±0.06 | 1.66±0.06 | 1.45±0.16 | 1.02±0.07 | 0.65±0.02 | 0.52±0.03 | 0.46±0.06 |
| Arginine | ND | ND | ND | ND | ND | ND | ND |
| Tryptophan* | ND | ND | ND | ND | ND | ND | ND |
| Valine* | 0.51±0.02 | 0.44±0.00 | 0.41±0.05 | 0.43±0.04 | 0.76±0.02 | 0.59±0.10 | 0.47±0.07 |
| Methionine* | 0.76±0.08 | 0.54±0.11 | 0.52±0.07 | 0.68±0.05 | 0.78±0.09 | 0.55±0.04 | 0.53±0.02 |
| Lysine* | 1.27±0.06 | 0.92±0.10 | 1.32±0.05 | 1.07±0.08 | 1.05±0.12 | 1.05±0.03 | 1.04±0.06 |
| Isoleucine* | 0.57±0.07 | 0.49±0.02 | 0.45±0.00 | 0.45±0.08 | 0.45±0.04 | 0.42±0.06 | 0.38±0.13 |
| Leucine* | 1.03±0.03 | 0.77±0.07 | 0.74±0.02 | 0.71±0.10 | 0.66±0.06 | 0.55±0.09 | 0.69±0.16 |
| Phenylalanine* | 0.88±0.14 | 0.82±0.04 | 0.78±0.05 | 0.67±0.12 | 0.62±0.10 | 0.53±0.07 | 0.36±0.06 |
| Threonine* | 1.35±0.07 | 1.16±0.11 | 1.00±0.01 | 0.92±0.13 | 0.60±0.06 | 0.60±0.02 | 0.56±0.07 |
| Total content | 12.51 | 10.46 | 11.32 | 10.30 | 9.70 | 8.16 | 7.18 |

Values represent the means ± SDs of triplicate samples. ND: not detected.

**Table S3** Mineral element contents of dried mycelia of different *V. volvacea* strains

| mineral elements (mg/kg) | M0 | M1 | M2 | M3 | M4 | M5 | M6 |
| --- | --- | --- | --- | --- | --- | --- | --- |
| Ca | 12.31±3.22 | 11.47±2.14 | 10.85±1.96 | 10.36±2.53 | 10.08±3.13 | 9.74±2.65 | 9.68±3.03 |
| Cu | 2.68±0.78 | 2.18±0.54 | 2.06±0.38 | 2.03±0.80 | 1.92±0.35 | 1.83±0.63 | 1.75±0.37 |
| Fe | 18.12±3.79 | 16.56±2.77 | 13.54±2.64 | 13.02±3.18 | 12.81±1.99 | 8.03±1.25 | 8.96±2.40 |
| K | 156.22±23.61 | 139.56±12.47 | 138.24±18.43 | 139.55±16.58 | 139.23±28.63 | 139.48±30.10 | 139.59±17.72 |
| Mg | 163.01±19.00 | 155.33±26.05 | 153.7±17.61 | 151.43±20.21 | 149.74±16.59 | 143.39±23.94 | 143.2±18.35 |
| Mn | 20.94±4.31 | 19.45±3.50 | 18.28±3.07 | 16.38±2.93 | 14.83±3.72 | 14.91±4.05 | 14.95±2.91 |
| Na | 16.5±2.64 | 12.73±3.61 | 12.72±1.78 | 12.72±1.26 | 12.03±3.58 | 10.51±2.02 | 10.69±1.63 |
| Zn | 8.27±1.05 | 4.18±0.53 | 3.52±0.80 | 3.46±0.93 | 3.06±0.47 | 3.27±0.69 | 3.29±0.55 |
| Total content | 398.05 | 361.46 | 352.91 | 348.95 | 343.7 | 331.16 | 332.11 |

Values represent the means ± SDs of triplicate sample.
